# Emerging Nano-Based Strategies Against Drug Resistance in Tumor Chemotherapy

**DOI:** 10.3389/fbioe.2021.798882

**Published:** 2021-12-07

**Authors:** Lei Cao, Yuqin Zhu, Weiju Wang, Gaoxiong Wang, Shuaishuai Zhang, Hongwei Cheng

**Affiliations:** ^1^ Department of Pathology, Quanzhou Women’s and Children’s Hospital, Quanzhou, China; ^2^ Department of Pathology, Qingyuan Maternal and Child Health Hospital, Qingyuan, China; ^3^ School of Pharmaceutical Sciences, Southern Medical University, Guangzhou, China; ^4^ State Key Laboratory of Molecular Vaccinology and Molecular Diagnostics and Center for Molecular Imaging and Translational Medicine, School of Public Health, Xiamen University, Xiamen, China

**Keywords:** tumor chemotherapy, drug resistance, tumor microenvironment, nanomedical strategy, combination therapy

## Abstract

Drug resistance is the most significant causes of cancer chemotherapy failure. Various mechanisms of drug resistance include tumor heterogeneity, tumor microenvironment, changes at cellular levels, genetic factors, and other mechanisms. In recent years, more attention has been paid to tumor resistance mechanisms and countermeasures. Nanomedicine is an emerging treatment platform, focusing on alternative drug delivery and improved therapeutic effectiveness while reducing side effects on normal tissues. Here, we reviewed the principal forms of drug resistance and the new possibilities that nanomaterials offer for overcoming these therapeutic barriers. Novel nanomaterials based on tumor types are an excellent modality to equalize drug resistance that enables gain more rational and flexible drug selectivity for individual patient treatment. With the emergence of advanced designs and alternative drug delivery strategies with different nanomaterials, overcome of multidrug resistance shows promising and opens new horizons for cancer therapy. This review discussed different mechanisms of drug resistance and recent advances in nanotechnology-based therapeutic strategies to improve the sensitivity and effectiveness of chemotherapeutic drugs, aiming to show the advantages of nanomaterials in overcoming of drug resistance for tumor chemotherapy, which could accelerate the development of personalized medicine.

## Introduction

The biggest barrier to cancer therapy is the inevitable emergence of drug resistance. Drug resistance can be divided into two categories according to the behind factors: primary and secondary (Longley and Johnston, 2005). Primary resistance indicates those factors present in the cancer cell or tissue itself before chemotherapy and reduces the efficacy of chemotherapy. However, because of various adaptive responses, such as increased expression of therapeutic targets and activation of alternative signaling pathways, secondary or acquired resistance can develop during therapeutic periods that are initially sensitive to cytotoxic agents (Lackner et al., 2012). Continuously activated proliferative signaling, inactivated growth suppressors, and activated metastasis factors are classical hallmarks of some tumors, which lead to drug resistance to chemotherapy. With the deepening of mechanism research, tumor heterogeneity, tumor microenvironment, and cancer stem cells (CSCs) were closely associated with the occurrence and development of drug resistance in chemotherapy. Besides, the characteristic of cell motility acquired from epithelial-mesenchymal transition (EMT), genetic factors of drug transport, especially the multiple drug resistance (MDR) is also considered to be vital factors for resistance development.

Considering the drug resistance is a complex mechanism, many dexterous solutions appeared in anticancer therapies to overcome drug resistance and increase the efficacy of treatment. Among these, nanomaterials as a promising drug delivery are being investigated for solving several forms of drug resistance (Markman et al., 2013; Da Silva et al., 2017). Compared with “free” drugs, nanomaterials have remarkable advantages containing higher bioavailability, slower drug release, lower drug usage, and better treatment outcomes. Statistically, there are more than 40 therapeutic nanomaterials approved for clinical use worldwide and at least 200 nanoparticles undergoing clinical trials (Schütz et al., 2013; van Der Meel et al., 2013; Bobo et al., 2016). The classical enhanced permeability and retention (EPR) effect in solid tumors promote the selective distribution of nanomaterials, further increase pharmacodynamics and reduce systemic side effects (Fang et al., 2011; Kobayashi et al., 2013). A possible concern is that the nanomaterials may be inclined to accumulate in drug disposal organs such as the liver and kidney, leading to severe organ toxicity. To avoid the side effects introduced by nanomaterials, researchers have developed lots of methods including changing the physicochemical properties of nanomaterial, PEG polymer chains modifications, and so on (Milla et al., 2012; Zhu et al., 2013). At present, more and more novel nanomaterial-based formulations are emerging in-clinic research, many of them win success especially in solving the therapeutic impediments in cancer treatment.

**SCHEME 1:**
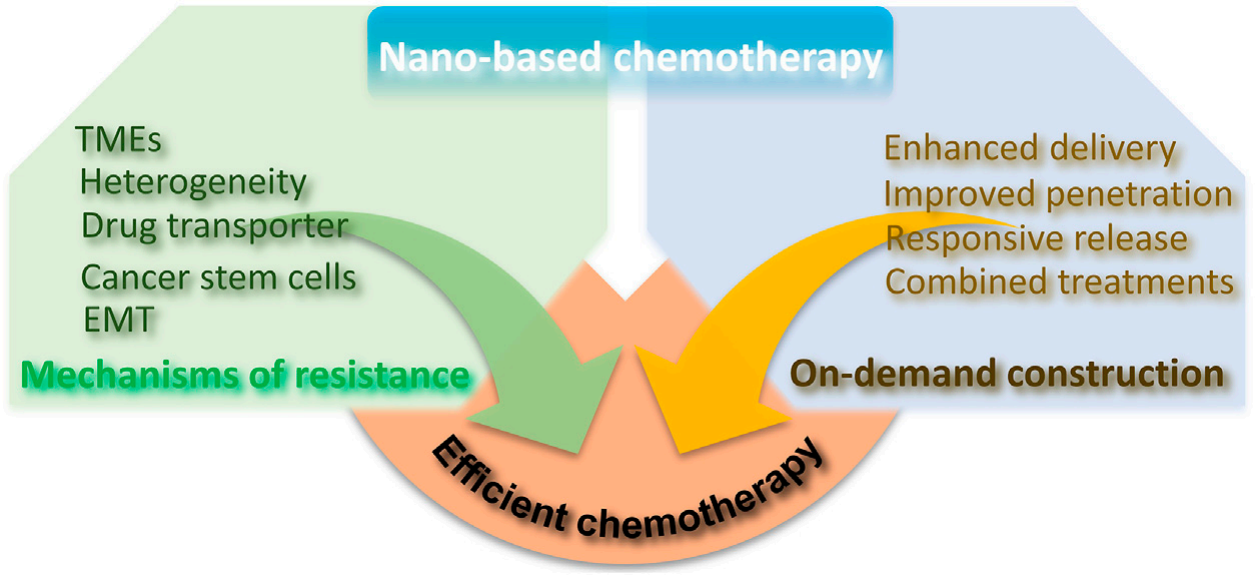
Illustration of chemotherapy resistance mechanism and the advantages of nano-based chemotherapy to achieve efficient chemotherapy.

Here, in this review, we mainly described the mechanisms of drug resistance and focused on recent innovation of dexterous and sophisticated nano-strategies in overcoming drug resistance, providing ideas for tumor chemotherapy and related combination therapies (Scheme 1).

## Mechanism of Drug Resistance

**FIGURE 1:**
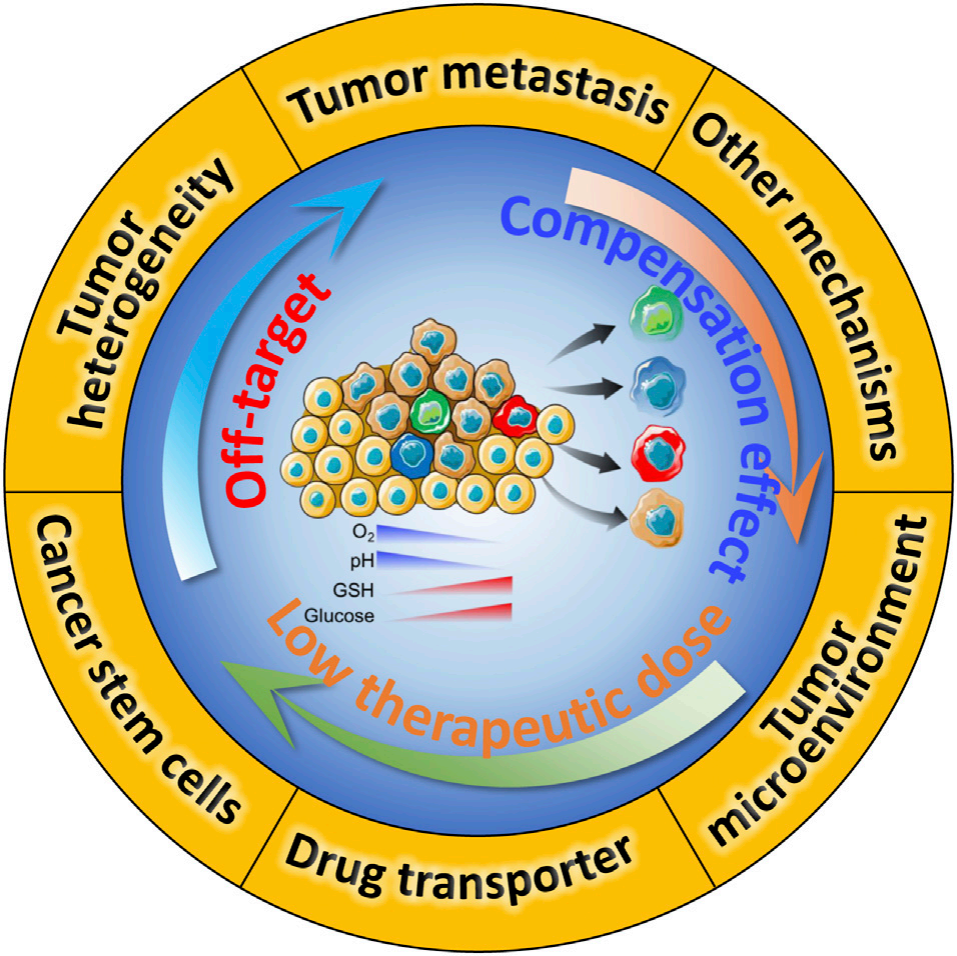
Schematic illustration of drug resistance in chemotherapy, and the underlying crosstalk between these mechanisms.

The mechanism of drug resistance at tumor tissue level is extremely complicated. Commonly, tumor heterogeneity, tumor microenvironment (TME), drug transporter and multidrug resistance, cancer stem cells (CSCs), epithelial-mesenchymal transition (EMT) and tumor metastasis are known as the major drug resistance factors (Figure 1), which contribute to the off-target effect in the practice of chemotherapy. And drug transporters-mediated drug efflux impairs the cellular chemotherapeutics delivery, leading to the low therapeutic dose (Cheng et al., 2018a; Fan et al., 2018; Ke et al., 2021). Besides, low pH, hypoxic tumor microenvironment, and other anti-apoptotic molecules could enhance the survival compensation effect (Li and Kataoka, 2021). Except for the five drug resistance reasons we mentioned, other resistant reasons containing gene mutations and genomic instability, epigenetic changes such as DNA methylation and protein acetylation, inhibition of apoptotic signaling and overexpression of anti-apoptotic molecules (Min et al., 2017; Vasan et al., 2019). To comprehensively explain the innovative design and prospects of nanomedicine in overcoming of tumor resistance, this review mainly focuses on several classic resistance mechanisms. Clinical practice shows serious resistance against chemotherapeutic drugs significantly impairs the therapeutical effect and contributes to poor prognosis for cancer patients. Therefore, full understanding the mechanism of drug resistance at the molecular level might bring novel treatment strategies, which in turn will lead to deeper and longer-lasting cancer treatments.

### Tumor Heterogeneity

Heterogeneity is a distinct characteristic of tumor tissues from normal tissues and is represented by different morphological profiles. The heterogeneity may cause discrepant distribution of tumor cell subpopulations across different regions of the primary tumor (spatial heterogeneity) or variations in the molecular makeup of a single lesion over time (temporal heterogeneity). Cancer cells have the powerful capacity of self-renewal and differentiation, which give rise to abundant tumor types causing heterogeneity of the tumors (Marusyk and Polyak, 2010; Dagogo-Jack and Shaw, 2018). The driving forces behind tumor heterogeneity contain heritable and non-heritable mechanisms, either as a result of a natural progression of tumor or exposure to pressures created by clinical interventions. A substantial fraction of heterogeneity commonly results from environmental factors during tumor development and is therefore non-heritable. Tumor heterogeneity can be regarded as a process of Darwinian evolution theory (Polyak, 2014; Zellmer and Zhang, 2014). Tumor heterogeneity affects drug sensitivity and provides fuel for drug resistance in cancer therapy. The example of drug response influenced by temporal heterogeneity comes from a cellular barcoding experiment results that resistant clones often arise from the selective expansion of pre-existing populations during drug treatment (Bhang et al., 2015). Spatial heterogeneity has a greater impact on initial drug treatment response. However, analyses involving single-site biopsy sampling might result in underestimation of the degree of spatial heterogeneity. Instead, noninvasive “liquid biopsies” extracted from patient blood are a promising strategy for addressing the shortcomings of tissue sampling (Nagrath et al., 2007; Chabon et al., 2016). Recently, single-cell sequencing technology is revolutionizing biomedical research. Using single-cell genomics enables to characterize tumor heterogeneity by quantifying the gene expression of thousands of individual cells (Villani et al., 2017).

### Tumor Microenvironment

The reprogramming of cellular metabolism is a common feature of cancer cells, one of which is known as the Warburg effect (Vaupel et al., 2019). Cancer cells would prefer to adopt aerobic glycolysis for growth rather than oxidative phosphorylation (OXPHOS), which strongly upregulate glucose uptake and aerobic glycolysis to increase intermediate glycolytic metabolites and the production of lactic acid (Lu et al., 2015). On the one hand, the glycolytic intermediates provide essential anabolic energy for cell proliferation and tumor growth. On the other hand, the increased by-product of lactic acid results in the accumulation of intracellular protons. The accumulation of protons in the intracellular environment activates the proton excludes (Na+/H+ exchanger, and carbonic anhydrase) and consequently changes the pH gradient in the tumor microenvironment (Izumi et al., 2003). This new biophysical setting profoundly influences the capability of drug uptake and pharmacological process in cancer cells. The lower pH value in tumor tissues gives rise to the protonation and neutralization of various chemotherapeutic drugs such as doxorubicin by preventing them from entering the targeted locus (Miraglia et al., 2005; Spugnini et al., 2015). Moreover, alteration of pH gradient easily promotes the formation of cytoplasmic acidic vesicles in tumor cells, which sequestrates chemical molecules in cell organelles eventually leading to the elimination of the drug through vesicle degranulation (Taylor et al., 2015).

Hypoxia is another common microenvironment feature in nearly all solid tumors. The uncontrolled proliferation of tumors limits the availability of oxygen supplied from the tumor vascular system, thus exposing them to low oxygen environments. On average, the oxygen level in tumor tissues is around 1–2% or below (McKeown, 2014). Hypoxia is also a key factor in cancer therapy resistance by regulating the tumor microenvironment (Rohwer and Cramer, 2011; Jing et al., 2019). The transcription factor hypoxia-inducible factor (HIF) usually serves as the major mediator of hypoxia response and is highly expressed in many cancers. Numerous evidence has shown that the inhibition of HIF-1α in cancer cells significantly enhanced sensitivity towards chemotherapeutic drugs (Brown et al., 2006; Liu et al., 2008). However, the underlying molecular mechanism of HIF-1α’s contributions to drug resistance is complicated, multiple, and specific in some tumor types. Some studies found that HIF-1 can induce the expression of the multidrug resistance 1 (MDR1) gene in a hypoxic tumor environment (Comerford et al., 2002). The membrane-located P-glycoprotein (P-gp) is the translation product of the MDR1 gene, which belongs to the family of ATP-binding cassette (ABC) transporters. Acting as a drug efflux pump, P-gp could decrease the intracellular concentration of chemotherapy agents such as paclitaxel, vinca alkaloids (Gottesman et al., 2002). Many chemotherapeutic agents such as 5-fluorouracil (5-FU), cisplatin, and DNA damage inducer triggering cell apoptosis largely depend on p53 pathway activation. Other important findings are that HIF-1 may function as an antagonist of p53-mediated cell death upon treatment with the chemotherapeutic agent (Hao et al., 2008; Rohwer et al., 2010). Besides, other reasons of HIF-1 contribute to chemotherapeutic drug resistance involving DNA damage repair, metabolic reprogramming, and immune microenvironment (Wirthner et al., 2008; Semenza, 2010; Vaupel and Multhoff, 2018).

### Drug Transporter and Multidrug Resistance

Drug transporters are integral membrane proteins that are involved in drug disposition by affecting absorption, distribution, metabolism, and excretion (ADME). Clinical pharmacokinetic drug-drug interactions (DDIs) studies have shown that transporters mainly participate in the drug ADME process together with metabolic enzymes (Zhang et al., 2008). Based on their structure and mechanism, membrane transporters can be divided into two major superfamilies: the ATP-binding cassette (ABC) and solute-linked carrier (SLC) superfamily (Giacomini et al., 2010; Nigam, 2015). One of the drug transporter so far received the greatest attention is ABC family transporters namely ABCB1, also known as P-glycoprotein or MDR1. Tumor cells can achieve resistance to a broad range of anti-cancer drugs via activating drug efflux pumps of membrane-resident P-glycoprotein (P-gp). These hydrophobic drug substrates such as anthracyclines, vinca alkaloids, taxane, epipodophyllotoxins, topotecan, as well as several pharmaceuticals agents are presented to the transporter directly from the lipid bilayer. Such self-defense effect can occur in ATP-dependent or ATP-independent manners regardless of the concentration gradients of drugs (Pérez-Tomás, 2006; Robey et al., 2018). Research has suggested that the expression of P-gp is usually upregulated in a variety of tumors including the colon, kidney, liver, and pancreas. When exposed to chemotherapy drugs, the mRNA levels of MDR1 can be rapidly induced in tumor cells (Abolhoda et al., 1999). A more important discovery is that transporter protects against cell death not only by forcing out drugs but also via direct interference with the caspase-dependent pathway, which is called as efflux pump-independent MDR effect (Pallis et al., 2002). For the above reasons, it seems that P-gp inhibition is a clever therapeutic way to reverse the MDR phenotype. However, many of the P-gp modulators were substrates for other transporters or metabolic enzymes resulting in unpredictable pharmacokinetic interactions. For this reason, considerable efforts should be made to develop agents specifically inhibiting P-gp function in MDR-mediated drug resistance research.

### Existence of Cancer Stem Cells

It is widely accepted that cancers develop from a small subset of stem cells with exclusive ability to self-renew and differentiate into the heterogeneous cancer cell types (Clarke et al., 2006; Beck and Blanpain, 2013). These intrinsic cancer stem cells or tumor-initiating cells constitute a population that is mainly responsible for cancer aggressiveness, drug resistance, and tumor relapse. There is no universal molecular feature of CSCs, although some cell-surface markers such as CD133, CD44 were associated with CSC phenotype in different types of tumors (Grosse-Gehling et al., 2013; Yan et al., 2015). Sometimes, it is assumed that the expression of these surface markers may reflect the CSC frequency rather than exact content in certain tumors. CSCs are rarely ranging from 1 per 1,000 to 1 per 100,000 in human tumors (Ishizawa et al., 2010). Nevertheless, when transplanted into the immunodeficient mice, these CSCs quickly enable the formation of secondary tumors. Chemotherapeutic drugs typically affect dividing cells, while CSCs are primarily quiescent cells with enhanced DNA repair mechanisms, always not influenced (Vitale et al., 2017). Besides, CSCs also express high levels of specific ABC drug transporters containing ABCB1, ABCG2, and ABCC1, which are known MDR genes in tumor cells, allowing for increased survival (Moitra, 2015). Although chemotherapy kills the majority of tumor cells, the resistant CSCs may still survive and expand the tumor again with self-renewing cells and differentiated offspring (Dean et al., 2005).

### EMT and Tumor Metastasis

Epithelial-mesenchymal transition (EMT) is a process that epithelial cells lose their apical-basal polarity and cell-cell adhesive properties, acquire migratory and invasive properties of mesenchymal cells. These undergoing EMT cells exhibit decreased expression levels of epithelial markers (E-cadherin) and increased expression levels of mesenchymal genes (N-cadherin and vimentin) (Lamouille et al., 2014). In most cases, EMT leads to cell morphological changes and the gain of stem cell-like features. EMT is extensively involved in multiple biological pathological processes, especially in many aspects of cancer progression, including tumor metastasis and drug resistance (Du and Shim, 2016; Du et al., 2017). In diverse types of cancers, these undergoing EMT cells frequently overexpressed ABC family transporters and showed drug resistance phenotype similar to CSCs (Mani et al., 2008; Jiang et al., 2017). Studies have demonstrated that the promoters of ABC transporters contain several binding sites for EMT-related transcription factors. Overexpression of transcription factors such as Twist and Snail could increase the promoter activity and expression of ABC transporters in cancer cells, causing drug resistance in cancer treatment (Saxena et al., 2011). Besides, matrix metalloproteinases (MMPs) are involved in the regulation of EMT and other metastasis-related molecules, the combination of specific inhibitors against MMPs is an effective strategy to prevent tumor metastasis and improve tumor chemotherapy (Chen X. et al., 2021).

## Nanomaterial Therapeutics Overcome Drug Resistance

Nanotechnology is initially defined as the “intentional design, characterization, production, and applications of materials, structures, devices, and systems by controlling their size and shape in the nanoscale from 1 to 100 nm range.” (N Engl J Med. 2010 Dec 16; 363 (25):2434–43) Nanotechnology has potential applications in medical applications because nanomaterials are similar in scale to biomolecules and systems, and can be designed to perform different functions. In cancer treatment, nanomaterials are mainly designed to aid in the transport of anticancer drugs through biological barriers. The aims of cancer nanomedicine are to use the properties and physical characteristics of nanomaterials for achieving more effective and safer cancer treatment (Nat Rev Cancer. 2017 Jan; 17 (1):20–37). In recent years, emerging evidence demonstrated that some nanomaterials have distinct advantages in reducing side effects and overcoming drug resistance. The following will focus on recent advancements in nanotherapeutic approaches to tackle drug resistance.

### Precise Individualized Strategies for Overcoming Tumor Heterogeneity

**FIGURE 2:**
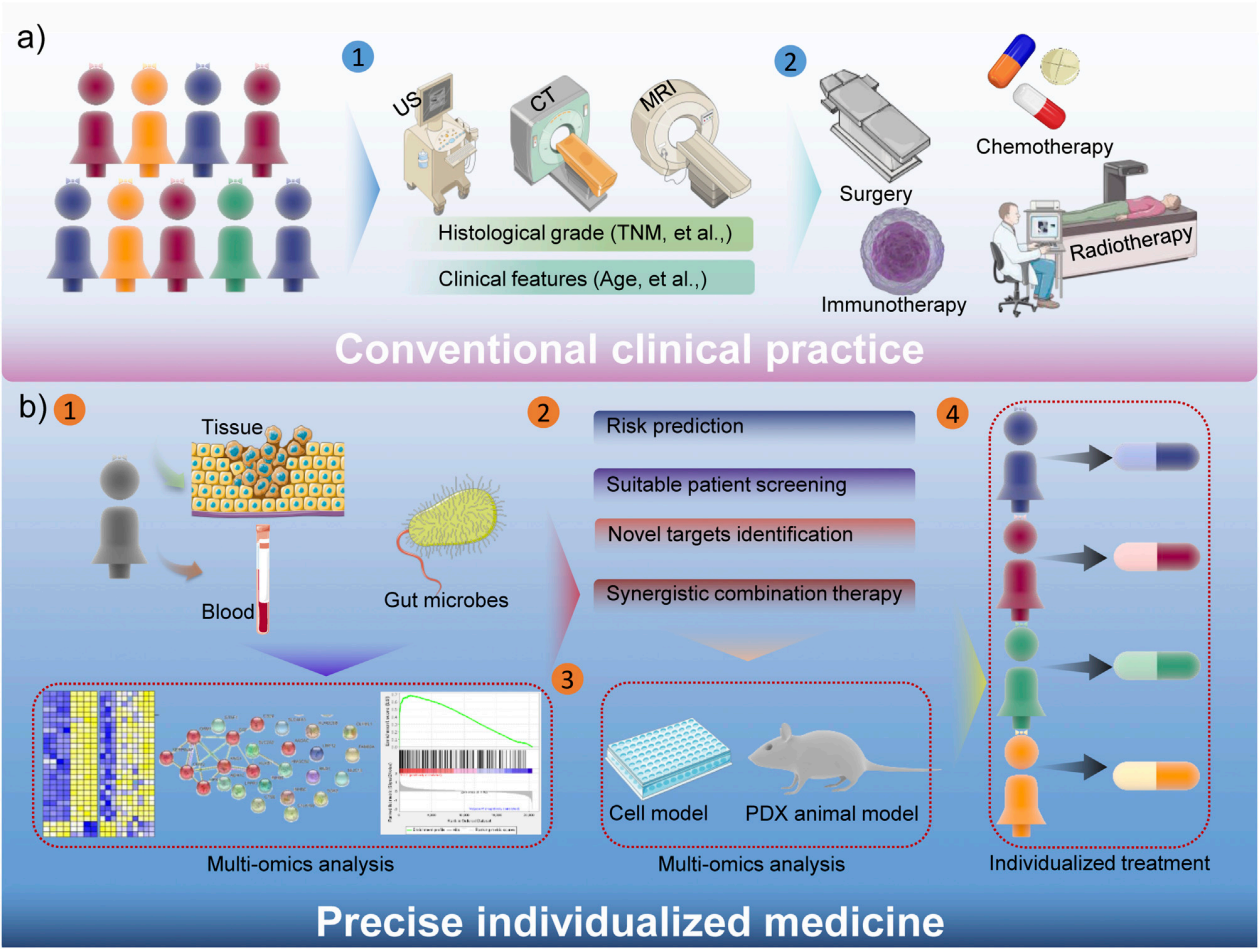
Schematic illustration of the advantages about precise individualized medicine.

Tumor heterogeneity is an important hallmark for some cancers, which contribute to the unsatisfactory treatment outcome (Liu et al., 2019). Traditional tumor treatments are mostly based on ultrasound (US), magnetic resonance imaging (MRI), computed tomography (CT) and other examinations to stage cancer patients, and then formulate treatment strategies based on histological grade (such as TNM staging, cancer thrombus formation, et al.) and some clinical characteristics of the disease, etc. (Lord et al., 2021). And then surgery, chemotherapy, radiotherapy, immunotherapy and other treatments could be chosen by median’s experience. Obviously, the consistency of conventional tumor treatments is poor, and this general treatments have a certain therapeutic effect on some patients, but due to the characteristics of tumor heterogeneity and other reasons, many patients have not benefited. The inhibitor against kirsten rat sarcoma virus (KRAS) oncogene is a good example. Due to the gene mutation of KRAS in many cancer patients, the clinical performance is not satisfactory (Kim et al., 2020). Another specific example is that sex also plays a critical role in the drug response, toxicity and other aspects (Hajipour et al., 2021). Therefore, precision medicine has received more and more attention in recent years, which could significantly compensate for the shortcomings of conventional strategies (Figure 2). Simply, patient’s sample-based multi-omics analysis could comprehensively demonstrate the underlying risk, suitable patients, novel drug targets, and promising combination therapies (Zhang and Yu, 2020). Not only that, precision medicine will also accelerate the process of clinical transformation, the cells and patient-derived xenograft (PDX)-based drug screening could enhance the efficacy of clinical trials (Wang et al., 2018a). Therefore, the precision medicine could guide the choice of clinical medication and treatment options, and more evidence also confirms that it is beneficial to improve the prognosis of cancer patients. Considering the better performance of nanomedicine in cancer therapy, the diverse functionality of nanomedicine remarkably facilitates precision nanomedicine, bringing revolutionary progress to tumor treatment.

### Exploiting Novel pH-Responsive Nanoparticles Based on Tumor Acidity

**FIGURE 3:**
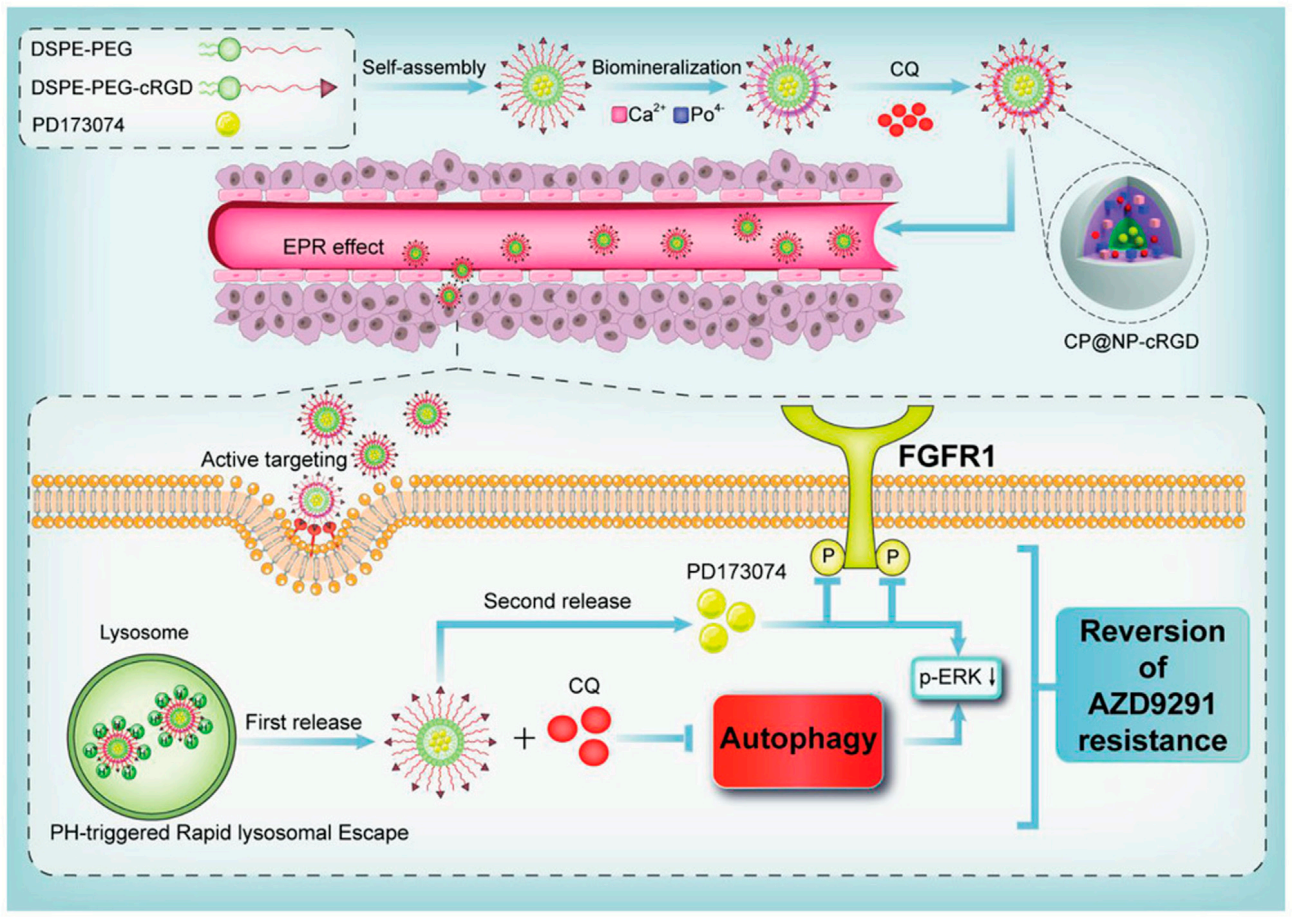
Schematic model of a pH-responsive shell-core nanoparticles to overcome AZD9291 drug resistance. Reproduced with permission (Gu et al., 2021). Copyright 2021, Wiley.

Recognizing that tumors tend to be acidic environments, a class of pH-responsive nano-structures is developed and such systems could enhance uptake by tumor cells through acidic microenvironment-triggered drug release. These nanoparticles were reported as following. Self-assembled PEG-detachable polymeric micelles DOX-PMs (Xu et al., 2019), zwitterionic charge-switchable polymers S-NP/DOX (Yuan et al., 2012), pH and redox dual responsive nanoparticle RPDSG/DOX (Kc et al., 2012), pH-sensitive DSF/PTX co-loaded micelles (Huo et al., 2017), dual-pH responsive BCP-DOX micelles (Mao et al., 2016), which were designed to combat chemotherapy resistance based on the low pH tumor microenvironment, and the results have implied that these pH-responsive micelles have significant potential for efficiently combating chemotherapeutic agents (DOX and PTX) resistance in cancer treatment especially in co-delivery applications (Yu et al., 2015; Wang et al., 2017). Liu et al. reported a pH-sensitive DSF/PTX co-delivery systems work in drug-resistant tumor cells model. Briefly, the micelles were composed of succinic anhydride-modified PTX (SA-PTX) and disulfiram (DSF), a P-glycoprotein (P-gp) inhibitor, was simultaneously encapsulated into the hydrophobic core of poly (ethylene glycol)-block-poly (l-lysine) (PEG-b-PLL). Such a drug carrier was characterized as follows: 1) the micelles remain negative surface charge under blood circulation, while quickly changes to positive under acidic tumor tissues owing to the hydrolysis of anhydride group. 2) the endocytosis effect increases the uptake of micelles. 3) the internalized carriers could quickly release DSF accompanied by deformation of micelles, and then PTX drugs are released in acidic organelles in a pH-dependent sustained manner. 4) Because P-gp was inhibited by DSF, the PTX could accumulate at a higher concentration and effectively kill resistant tumor cells (Huo et al., 2017). Another good example is the combined PD173074 chemotherapeutics and chloroquine (CQ) to block the autophagy and fibroblast growth factor receptor 1 (FGFR1) mediated AZD9291-resistance (Figure 3). In details, a pH-sensitive nanoparticle system with shell-core structure was designed to encapsulate FGFR1 inhibitor PD173074 and autophagy inhibitor CQ to overcome the resistance of tyrosine kinase inhibitors (TKIs) AZD9291 in non-small cell lung cancer (NSCLC). FGFR1 signaling was described to contribute to EMT-associated acquired resistance of EGFR-TKI in NSCLC treatment. Therefore, NSCLC cells with EGFR-TKI resistance is sensitive to FGFR1 inhibitors (Wang et al., 2016). Further, the FGFR1 signaling could regulate cell autophagy, and autophagy inhibition is beneficial to enhance the anticancer effects of FGFR1 inhibitor (Lee et al., 2016). The novelty of this study is the application of pharmacological effects for innovative nanotheranostic. Furthermore, the biomineralization with CaP favour the nanoparticles with pH-sensitive to achieve the lysosome escape and realize the pH-responsive drug release, which could be applied by the low pH tumor microenvironment. The specific pH-activation drug release is fully studied, and many combined strategies were also developed based on other tumor microenvironments, such as higher glucose, low oxygen and higher Glutathione (GSH) concentration (Li et al., 2013; Li et al., 2017; Cheng et al., 2021). These pH-responsive nanoparticles not only can realize the escape of lysosome and enhance the pharmacological concentration, also achieve the responsive drug release to decrease the toxic side effects.

### Nanoparticles Targeting the Hypoxic Tumor Microenvironment

**FIGURE 4:**
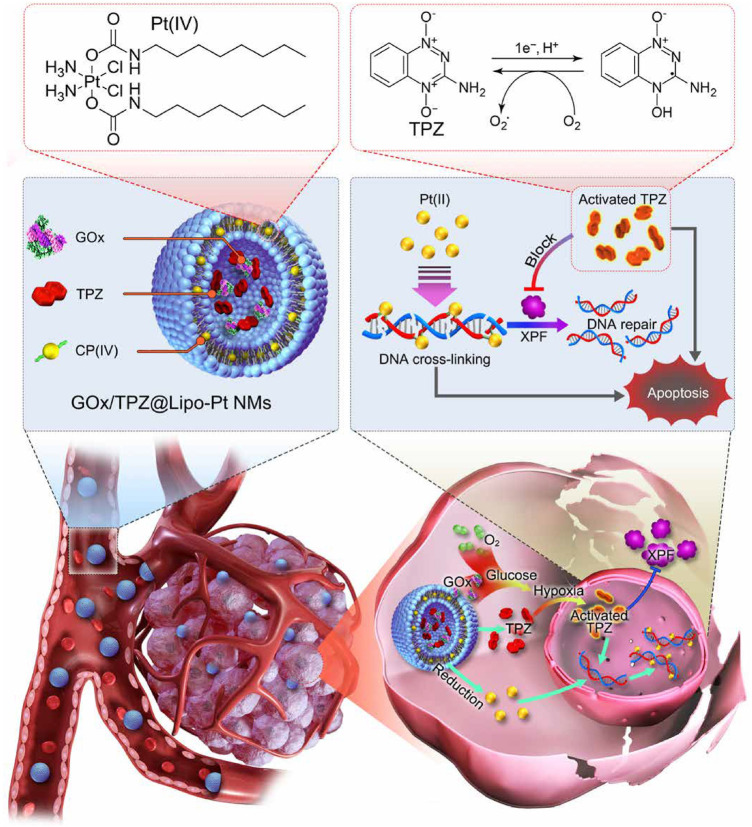
Schematic illustration of the GOx/TPZ@Lipo-Pt nanomaterial and hypoxia-induced reversal of cisplatin resistance. Reproduced with permission (Chen J. et al., 2021). Copyright 2021, American Chemical Society.

Evidence has revealed that hypoxia in the tumor microenvironment has a crucial role in the clinical resistance of chemotherapy through a variety of signal transduction pathways and molecular changes (Pastorek and Pastorekova, 2015; Wang D. et al., 2021; Xu et al., 2021). The development of advanced nanomaterial delivery methods for reversing the resistance of hypoxic tumors is therefore of great interest but remains a considerable challenge. In general, strategies modulating hypoxia via nanomaterials that have been investigated can be divided into three categories including countering hypoxia, disregarding hypoxia, and exploiting hypoxia. The countering hypoxia strategy is a traditional targeting method. For example, the recent study designed a versatile tumor hypoxia-directed nanoparticle loaded with Acetazolamide (ATZ) specifically targeting the marker of tumor hypoxia marker carbonic anhydrase IX (CA IX) for reversing Sorafenib resistance in RCC treatment (Alsaab et al., 2018). Based on the biocompatibility and oxygen dissolving ability of perfluorocarbon (PFC), others developed an innovative nano-PFC as an oxygen shuttle for ultrasound-triggered tumor-specific delivery of oxygen (Song et al., 2016). This kind of efficient oxygen-enhanced nanomaterial may also provide a promising strategy to overcome the hypoxia-associated resistance in cancer treatment (Tian et al., 2017). Interestingly, some material scientists tend to explore and make use of the hypoxic feature in drug-resistant tumor cells that is called as exploiting hypoxia strategy. Numerous studies have indicated that hypoxia-triggered DNA damage, mitochondrial activity, autophagy, and drug efflux are treatment obstacles in platinum-based clinical chemotherapy. For example, the xeroderma pigmentosum group F (XPF), an DNA self-repairing protein could be regulated by hypoxia, which causes the acquired resistance of cancer cells to cisplatin (Angew Chem Int Ed Engl. 2016 Dec 12; 55 (50):15564–15568). Recently, researchers proposed a novel nano-chemotherapy strategy that is to build a liposome nanodrug containing glucose oxidase (GOx), tirapazamine (TPZ), and platinum (IV) prodrug (Chen J. et al., 2021). The nanodrug can not only be fully utilized but also further aggravate intracellular hypoxia of cisplatin-resistant tumor cells, thus fully activating TPZ drug activity. Activated TPZ effectively inhibited the expression level of XPF protein promoting DNA repair in tumor cells, thus achieving synergistically enhanced anti-tumor therapy with platinum drug (Figure 4).

### Typical Nanocarriers for Overcome of Drug Pump Efflux

With the development of nanotechnology, more and more new-style nanostructures were discovered. Among these, gold nanoparticles (AuNPs) are being widely used to deliver small molecules due to their unique optical properties and surface plasmon resonance effect. And PEG can be attached to the surface of gold nanoparticles to increase the stability and circulation time of chemotherapy drugs. Moreover, the surface plasmon band (SPR) of the nanoparticle will be modified by changing the shape of AuNPs from spheres to rods. One of the advantages of gold nanorods is the shift of the SPR allows for near-infrared (NIR) absorption at the cross-sections permitting a deeper penetration into living tissues (Arvizo et al., 2010). Many results have demonstrated these AuNPs or gold nanorods could be used for the circumvention of drug resistance (Song et al., 2017; Wang et al., 2018b). One notable work is the study that Vishwakarma SK et al. developed a stable colloidal suspension of sorafenib-gold nanoconjugate (SF-GNP) in an aqueous medium for reverting the drug resistance in HCC cells in a 3D model system. Owing to the potential of highly biocompatible, SF-GNP nanoconjugates significantly reduced the growth and proliferation of SF resistant tumor cells with very least or no side effect after intra-peritoneal administration of SF-GNP nanoconjugates in animals (Vishwakarma et al., 2017).

**FIGURE 5:**
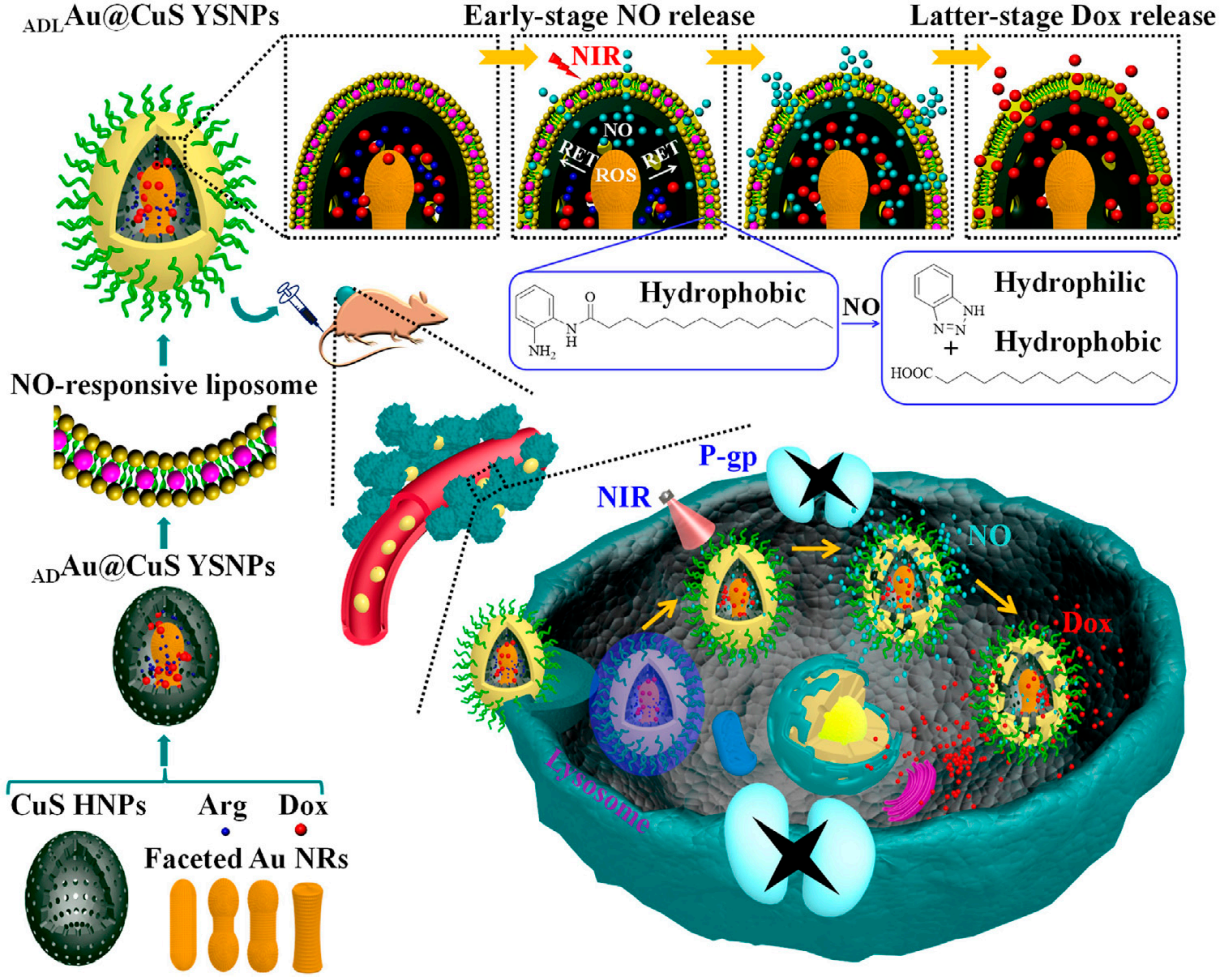
Illustration of the NO and Dox programmable released ADLAu@CuS YSNPs in MDR cancer therapy. Reproduced with permission (Wang L. et al., 2019). Copyright 2019, American Chemical Society.

Except for gold nanoparticles, another representative nanomaterial is the nitric oxide (NO)-stimulated nanosystem for multidrug resistance cancer therapy. This system reversing the MDR effect attribute to the principle that gaseous signaling molecules nitric oxide (NO) can be used as ABC transporter inhibitors to downregulate the expression level of P-gp, thus creating a favorable microenvironment for the treatment of drug-resistant cancer cells (Pieretti et al., 2020). Interestingly, Wang et al. designed a sophisticated nanosystem containing NO-responsive liposome, encapsulate l-arginine (l-Arg)/Dox-loaded gold@copper sulfide yolk-shell nanoparticles (ADAu@CuS YSNPs) to programmable release Dox in Dox-resistant cancer. Various faceted Au NRs were embedded in CuS HNPs to optimize Au@CuS YSNPs. The finally formed ADLAu@CuS YSNPs can convert L-Arg into NO upon NIR laser irradiation, leading to hydrolysis of o-phenylenediamine-containing lipid and programmable release of NO and Dox. Once internalized into MCF-7/ADR cells, the early stage NO release of ADLAu@CuS YSNPs can inhibit P-gp expression and create a favorable microenvironment for the latter stage Dox accumulation, beneficial for MDR cancer therapy (Wang L. et al., 2019). This NO and Dox sequential release of ADLAu@CuS YSNPs presenting a potential direction for drug-resistant cancer therapy (Figure 5).

The combination of chemotherapy and photothermal therapy is a promising strategy for cancer treatment. Despite the advantages in overcoming drug resistance, more studies have demonstrated the promising applications of these gold nanoparticles or NO-releasing nanomaterials in combined chemo-photothermal therapy (Zhang et al., 2016; Wei G. et al., 2019; Wang J. et al., 2021).

### Co-Delivery Nano-Formulations for Reversal of Resistance Mechanism

**FIGURE 6:**
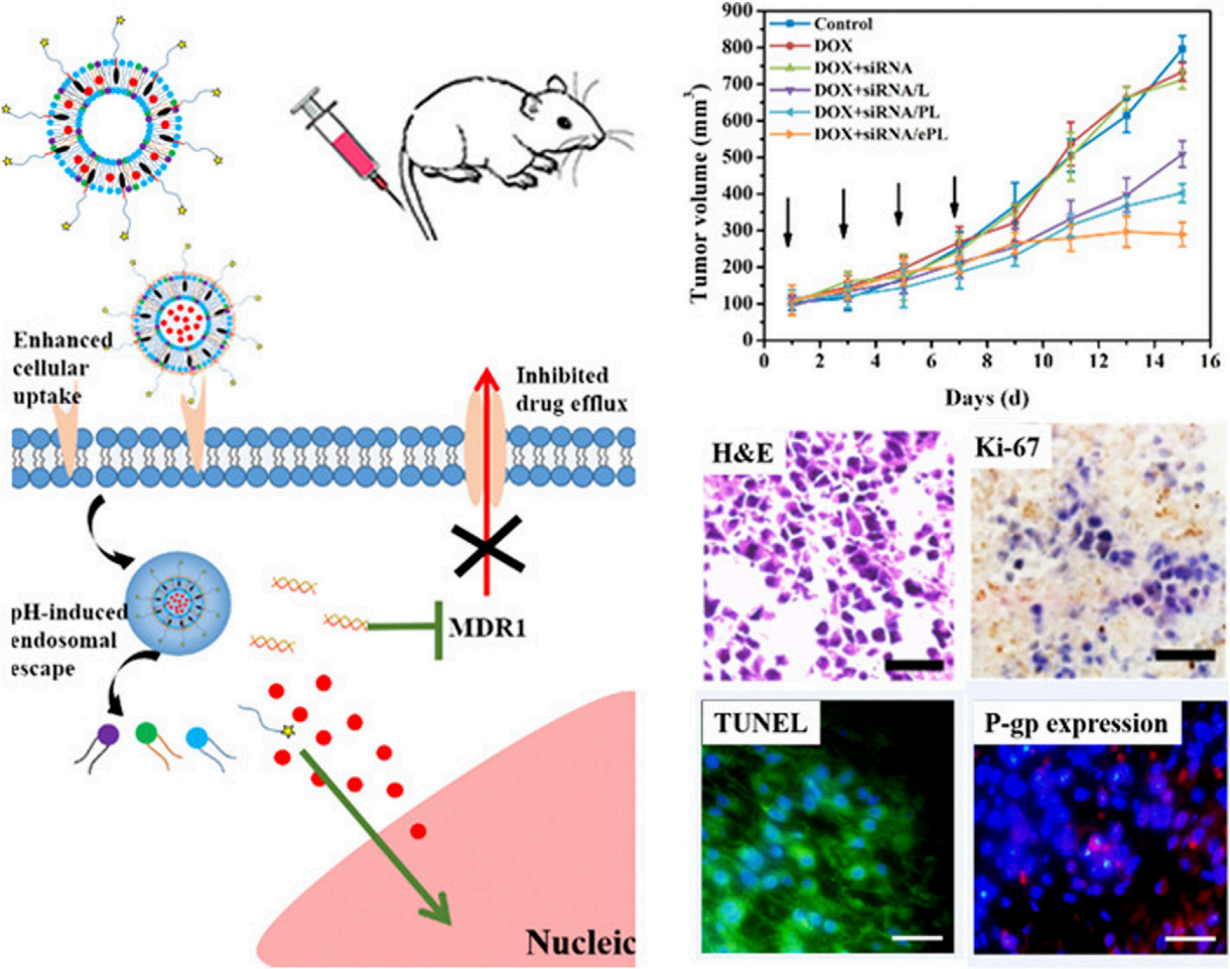
Schematic illustration of multifunctional DOX + siRNA/ePL lipoplexes to overcome MDR effect. Reproduced with permission (Zhang et al., 2018). Copyright 2018, American Chemical Society.

The co-delivery system of chemo-pharmaceutical agents and siRNA is an excellent nano-therapeutic strategy for the effective killing of tumor cells (Xiao et al., 2017; Li et al., 2019). Since the major mechanisms of drug resistance are the overexpression of drug-resistant associated genes such as drug transporters, one approach for overcoming drug resistance is to use the co-delivery strategy that utilizes small interfering RNA (siRNA) to silence the expression of drug-resistant associated genes together with chemotherapeutic drugs. Reports have demonstrated that these co-delivery nanocarrier systems still potently reverses the multi-drug resistance effect in tumor tissues even under hypoxic and acidic conditions (Butt et al., 2016; Zeng et al., 2017; Marson et al., 2019; Joshi et al., 2020). The expression of the multidrug resistance 1 (MDR1) gene is a major obstacle that hinders the treatment of numerous cancer. Notably, the targeted resistant genes loaded in nanoparticles not only contain the MDR1 (P-gp) gene but also other genes including the STC2 gene in liver cancer, K-Ras gene in lung cancer, and Bcl2 gene in breast cancer (Jeong et al., 2017; Wen et al., 2017; Cheng et al., 2018b). Recently, Zhang et al. designed a multifunctional pH-sensitive drug delivery system loaded with siRNA and DOX for drug-resistant breast cancer treatment. The nanocarrier is comprised of EphA10 antibody-conjugated pH-sensitive doxorubicin (DOX), co-loaded with MDR1-siRNA liposome (shortened as DOX + siRNA/ePL). Results from the intracellular study indicated that DOX + siRNA/ePL possessed the ability for incremental cellular uptake and rapid endosomal escape in a time-dependent manner. Meanwhile, the animal experiments suggested that DOX + siRNA/ePL could inhibit the proliferation, induce apoptosis, and downregulate the P-gp expression *in vivo* (Zhang et al., 2018). Altogether, DOX + siRNA/ePL was expected to be a suitable co-delivery system for overcoming drug-resistant effects in breast cancer treatment (Figure 6).

### Enhanced Drug Delivery by Antibody-Modified Active Targeting

**FIGURE 7:**
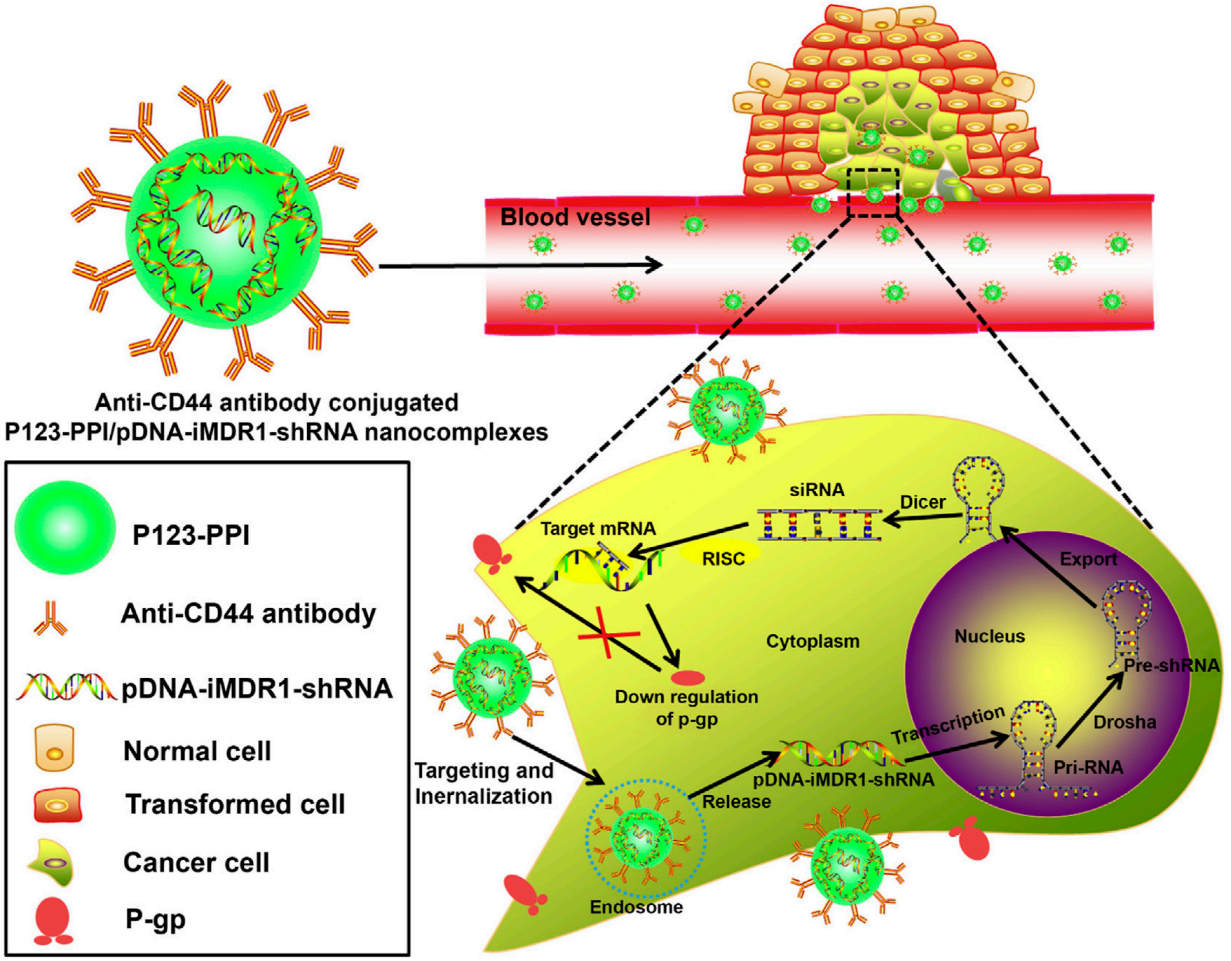
Schematic representation of the gene-silencing system of anti-CD44-P123-PPI/pDNA-iMDR1-shRNA nanocomplexes. Reproduced with permission (Gu et al., 2015). Copyright 2015, ELSEVIER.

The low drug therapeutic dose is an important factor of acquired resistance in cancer treatment, efficient drug delivery could improve the pharmaceutic effect and prevent the occurrence of drug resistance. Sorafenib (SFB) is a common chemotherapeutical drug targeting vascular endothelial growth factor receptor (VEGFR), which is universally overexpressed in many tumors (Gounder et al., 2018). The therapeutic efficacy of sorafenib (SFB) in the clinic was greatly limited due to its short half-life as well as drug resistance (Wu et al., 2019). To solve these problems, Gan et al. developed a novel SFB-loaded polymeric nanoparticle for targeted therapy of liver cancer. This nanoparticle was fabricated from self-assembly of biodegradable block copolymers and drug SFB, followed by conjugating the anti-GPC3 antibody, referred to NP-SFB-Ab (Gan et al., 2018). Results have shown that NP-SFB-Ab greatly inhibits the tumor growth of xenograft tumors without obvious side effects. Other antibody-conjugated nanoparticles include HER2-antibody conjugated nanocarrier for multi-drug-resistant breast cancer therapy, ICAM-1 as well as Trop2 antibody-mediated nanoparticles for triple-negative breast cancer (TNBC) treatment (Vivek et al., 2014; Son et al., 2018; Wang M. et al., 2019). Gu et al. explored Pluronic P123-conjugated polypropylenimine (PPI) dendrimer (named as P123-PPI) to deliver shRNA against MDR1 in breast cancer cells (Gu et al., 2015). In detailed, anti-CD44 monoclonal antibody was also included to form nanocomplex, achieving the efficient gene delivery, enhanced tumor targeting and longer blood circulation. Based on the above design, the nanocomplexes significantly inhibited the P-gp expression, simultaneously drug resistant cells showed the increased cellular adriamycin (ADR) accumulation (Figure 7).

### Combined Strategies in Reversal of Drug Resistance

The combinatorial strategy of chemo-chemo, chemo-immune, and chemo-photothermal treatments, usually called “cocktail therapy”, have been reported to be effective solutions to overcome drug resistance. For example, Zhang et al. developed an iRGD peptide-modified lipid-polymer hybrid nanosystem (LPN), co-loaded with paclitaxel (PTX) and tetrandrine (TET). As expected, the PTX + TET/iRGD LPNs significantly suppressed P-gp expression, promoted ROS production and apoptosis in PTX-resistant tumors (Zhang et al., 2017). Like “Domino-effect”, the chemotherapy combined with immunotherapy could amplify the efficacy of anti-tumor treatment. Studies have demonstrated that many advanced nanoparticles are competent to break through barriers in combinatorial chemo-immune treatment (Wei X. et al., 2019; He et al., 2021; Yan et al., 2021). Notably, the latest study reveals the phenomenon that some nanomaterials possess the inherent biological effect on immunotherapy. These nonfunctional nanomaterials can overcome tumor resistance to PD-1 antibody and sensitize the therapeutic effect of PD-1 antibody, thus presenting a promising prospect in chemo-immune therapy (Sun et al., 2021).

**FIGURE 8:**
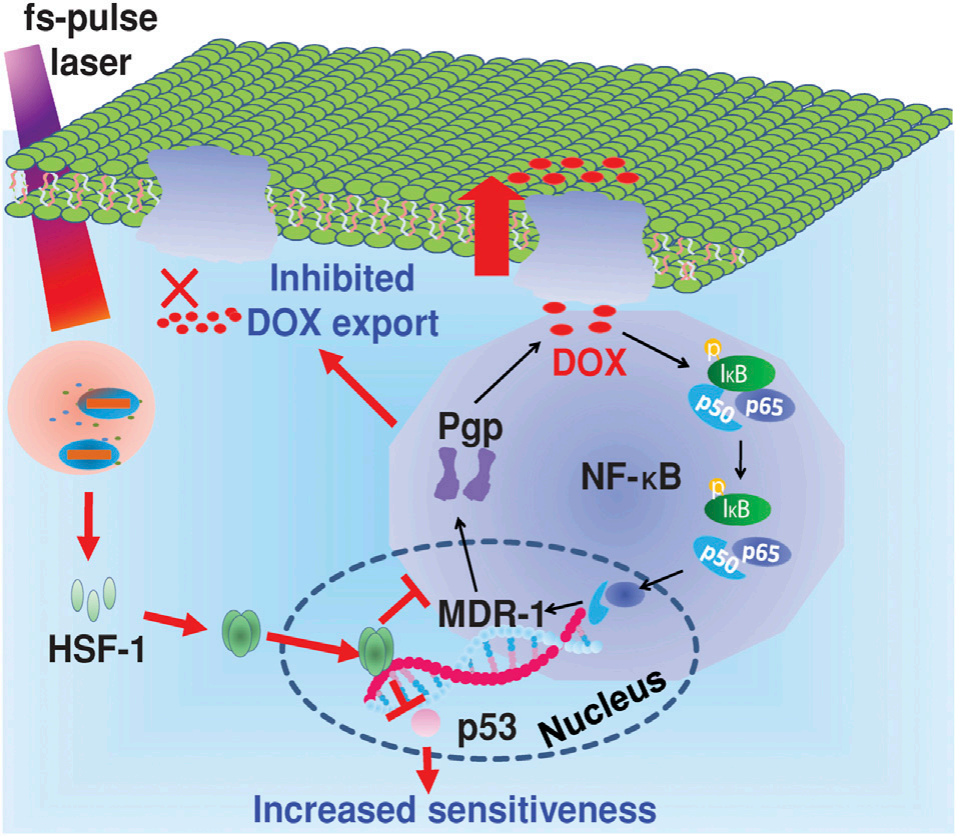
Mechanism of the reversal of drug resistance in cancer cells under fs-pulse laser irradiation. Reproduced with permission (Wang et al., 2014). Copyright 2014, Wiley.

Herein, the following picture simply illustrated a case that chemo-photothermal treatment applied in overcoming drug resistance (Figure 8). Wang et al. found that irradiation of Au@SiO2 resistant cells with femtosecond pulses at a lower intensity than photothermal therapy produced a unique photothermal effect, which induced intracellular heat shock factor (HSF-1) expression, inhibited the expression of p-glycoprotein (P-gp) on the cell membrane, and the NF-κB pathway. In addition, it can successfully induce the degradation of p53 protein mutated in drug-resistant cells, sensitize to chemotherapy drugs, and finally effectively overcome tumor drug resistance. Therefore, fs-pulsed laser irradiation provided a novel and promising strategy to combat drug resistance with the aid of a multifunctional nanocarrier (Au@SiO2) and triggered photothermal effects (Wang et al., 2014).

### Interaction of Chemotherapeutics and Metastasis-Related Inhibitors

**FIGURE 9:**
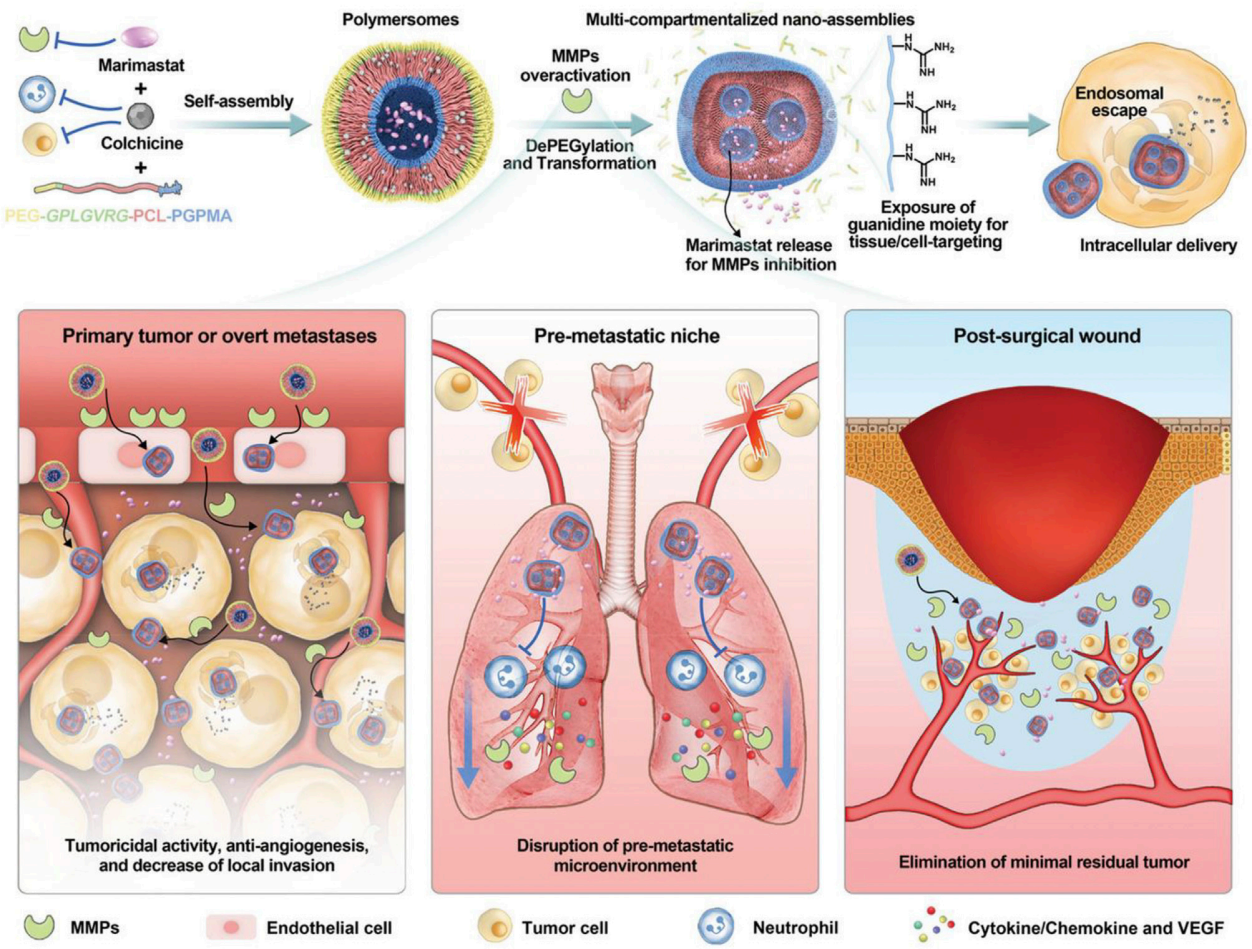
Schematic illustration of MMPs inhibition to eliminate tumor metastasis. Reproduced with permission (Li et al., 2021). Copyright 2021, Wiley.

Tumor metastasis is a major risk factor for poor prognosis of cancer patients, which is the most concerned issue in surgical treatment. Moreover, chemotherapy has little benefit in the treatment of multiple metastasis (He and Shi, 2014). Herein, the efficient inhibition of tumor metastasis is an urgent work to enhance the pharmaceutical effect of chemotherapeutics. Li et al. reported an enzymatically polymer to simultaneously deliver colchicine chemotherapeutics and marimastat, which is a potent MMPs inhibitor (Figure 9). Based on the efficient drug delivery, this nanosystem could disrupt the microtubules of tumor cells by colchicine, and simultaneously prevent tumor metastasis microenvironment, which could reduce the primary post-surgical recurrence and distant metastasis significantly (Li et al., 2021). To be noted that, MMPs are commonly activated in some tumor tissues, some nanosystem were developed to release chemotherapeutics in MMP-responsive manner (Yao et al., 2018; Vaghasiya et al., 2020), displaying an enhanced chemotherapeutical response.

## Conclusion and Perspectives

Tumors are composed of mutually interacting tumor cells and tumor microenvironment. Consequently, internal cancer cells along with external environmental factors, extracellular matrix (ECM), and immune cells eventually determine the tumor fate. Tumor heterogeneity, the existence of CSCs, and the occurrence of EMT process in tumor cells are the inner driving force in chemotherapy resistance. However, alterative external pressure including hypoxia, acidic environment, and tumor-associated macrophage (TAM) directly affect drug kinetics and favor the selection of more malignant, therapy-resistant tumor cell subpopulations. Since the complexity of tumor heterogeneity and microenvironment, the causes of drug resistance may differ from individual patients and within a single tumor. Thus, the therapeutic strategies based on a single and fixed chemotherapy drug are inevitably suboptimal. In contrast, flexible and diverse drug delivery materials provided a window into the availability of better treatment. What’s more, multiple nanoparticles applied in chemotherapy regimens could be effectively combined with photothermal therapy, immunotherapy, and CRISPR-mediated gene edit, thus synergistically acquiring better therapeutic efficacy in cancer treatment (Cheng et al., 2020). Considering the complexity and the heterogeneity of tumors, it is necessary to distinguish which nanotechnology strategy is most likely effective from a given context. This is just like the targeted therapy in individual patients or as we usually called precision medicine (Mitchell et al., 2021). Of course, one of approaches is to replace the traditional small molecule drugs with more smart drugs such as anti-cancer peptides (ACPs). Compared with chemical drugs, peptide-based drug delivery strategy has obvious advantages in cancer treatment, especially in overcoming drug resistance. In addition, the conjugation of peptides to nanoparticles results in advanced materials for treatment of cancer, whose properties can be adjusted to maximal efficacy for a given application (Scarberry et al., 2008; Olson et al., 2010).

In recent years, immunotherapy-combined nanoformulations have been developed based on studies of tumor escape mechanism, transforms immunosuppressive tumors into immunostimulatory phenotypes and overcomes the pathways leading to tumor escape. The therapeutic strategy has the potential to induce durable antitumor immune responses in hematologic and solid malignancies and thus has become treatment algorithms for multiple tumor types (Bhatia and Kumar, 2014). Cancer immunotherapy expands potential targets from tumors to the entire immune system, which is an area worthy of further exploration. Therapeutic approaches to manipulate various aspects of the immune system have been widely investigated including immune checkpoint inhibitors, CAR T cell adoptive immunotherapy, oncolytic viruses and vaccines (Yang, 2015). Also, increasing efforts are attempting to employ combination therapy to engage different parts of the immune system, implying the importance of this holistic approach. Through integrating with immunotherapy, nanomedicine improves the delivery of immune stimulators to create a close dialogue between tumors and immune system, which provides unique opportunities to complement each other. In turn, immunotherapy may weaken the effect of biological barriers for nanomedicine, and sometimes may not require large-scale targeted tumor killing activity because activating a small number of immune cells in the tumor or surrounding tissue can spread an immune response (Li and Kataoka, 2021). Compared with solely chemotherapeutic method, immuno-oncological or immuno-chemical nanomedicine strategies have the great potential to improve the efficiency toward tumor therapy. In our personal view, tumor immunotherapy can be an excellent way to overcome chemotherapeutic drug resistance because this strategy directly targets the tumor microenvironment rather than the heterogeneous tumor cells to achieve efficacy. Furthermore, nano-immunotherapy strategy could also prevent the function of cancer stem cells (CSCs), which could significantly enhance the sensitivity of chemotherapy and tumor recurrence (Lang et al., 2019). All of these evidence shows the promising outcome of nanotherapeutics with combination of immunotherapy and chemotherapy.

It is challenging to design nanomaterials to share all desirable characteristics for overcoming all barriers. A concrete design should mainly depend on the specific drug cargo and tumor target. An ideal drug delivery system should adequately protect drug cargo, minimize nonspecific interactions with biological species, and allow efficient aggregation to target sites. In addition, the drug delivery system, once accumulated in tumor tissue, should provide sufficient penetration to deliver the drug throughout the tumor tissue, resulting in mass apoptosis of tumor cells (Li et al., 2014; Li et al., 2015; Li et al., 2021). For a long time, we have been tried to design more complex nanomaterials in many circumstances. However, the design itself is flawed to some extent. Besides, exploring the function of nanomaterial itself *in vivo* and its interaction with biological system at the tissue, cellular, and molecular levels are always ignored. We strongly suggest that researchers should pay more attention on the material itself biological functions and its pharmacokinetics *in vivo*. What’s more, many efforts should be made to provide definitive evidence for its functional mechanism instead of creating many new complicated nanoparticle systems for similar concept.

At present, nanoparticles are trying to open a new era for tumor treatment. Though nanomedicine is still at an early stage, the reported results in cancer treatment especially in reversing drug resistance are surprising, and importantly, these advanced antitumor concepts are of great references for chemists, materials scientists, biologists, and may provide new insights into cancer and even a breakthrough in other fields.
